# Multimodal Pharmacological Assessment of *Arenga porphyrocarpa* Palm Leaves and Stems Extracts: Insights From In Vivo, In Vitro, and In Silico Approaches

**DOI:** 10.1002/fsn3.71178

**Published:** 2025-11-19

**Authors:** Jahid Hasan Azad, Fowzul Islam Fahad, Koushik Barua, S. M. Asadul Karim Azad, Safaet Alam, Fahmida Tasnim Richi, Rasel Khan, Syed Mohammed Tareq, Muhammad Mutasim Billah, Md. Sohel Rana, Mohammad Nazmul Islam

**Affiliations:** ^1^ Department of Pharmacy International Islamic University Chittagong Chittagong Bangladesh; ^2^ Department of Pharmaceutical Chemistry, Faculty of Pharmacy University of Dhaka Dhaka Bangladesh; ^3^ Chemical Research Division, BCSIR Dhaka Laboratories Bangladesh Council of Scientific and Industrial Research Dhaka Bangladesh; ^4^ Department of Pharmacy University of Asia Pacific Dhaka Bangladesh; ^5^ Department of Pharmacy Jahangirnagar University Dhaka Bangladesh

**Keywords:** anti‐depressant, anti‐oxidant, anxiolytic, *Arenga porphyrocarpa*, cytotoxic, GC–MS analysis, molecular docking, thrombolytic

## Abstract

*Arenga porphyrocarpa* palm tree roots and buds are used to treat urinary incontinence, fever, appetite loss, and as a diuretic. Our study aimed to evaluate the neuropharmacological, antioxidant, cytotoxic, and thrombolytic potentials of *A. porphyrocarpa* leaf and stem extracts through in vivo, in vitro and in silico studies. Different fractions of *A. porphyrocarpa* leaves and stems were investigated for anxiolytic and anti‐depressant efficacy through EPM and HBT and TST and FST tests. The anti‐oxidant assessment was assessed by using DPPH and reducing power assays, while thrombolytic efficacy was analyzed by clot lysis technique. Computational methods like in silico molecular docking, pass prediction and ADME/T were applied to evaluate several pharmacological properties of 15 selected compounds from GC–MS analysis of plant extract. Both doses of the plant extract fractions (200 and 400 mg/kg) showed dose‐dependent anxiolytic and antidepressant‐like effects in mice, with the 400 mg/kg dose found to have highly significant (*p* < 0.001) effects. Among the fractions, the chloroform fraction of *A. porphyrocarpa* leaves (CFAPL) exhibited the strongest antioxidant activity, as indicated by its DPPH scavenging potential (IC₅₀ = 128.94 μg/mL), and also demonstrated the most effective clot lysis. In silico analysis further revealed that several bioactive compounds had strong binding affinities to target proteins, including isobutyric acid 2,2,2‐trichloroethyl ester (anxiolytic activity), 2‐cyclobutene‐1‐carboxamide (antidepressant activity), 2‐pyridinecarboxylic acid (antioxidant activity), propionic acid 3‐(isobutylthio)‐ (cytotoxic), and 2‐propanone 1‐hydroxy‐ (thrombolytic activity). Ongoing investigations indicate that *A. porphyrocarpa* is a promising candidate for the treatment of neuropsychiatric and cardiovascular disorders; however, further cytotoxicity studies and confirmation of the specific bioactive compounds responsible for its biological activities are needed.

## Graphical abstract

Arenga porphyrocarpa leaf and stem extracts demonstrated significant neuropharmacological, antioxidant, and thrombolytic potentials. GC–MS profiling identified 51 bioactive constituents, while molecular docking revealed strong affinities toward key biological targets underlying these pharmacological effects. These integrated findings suggest that A. porphyrocarpa contains diverse phytoconstituents with multi‐target pharmacological potential, underscoring its therapeutic promise in mitigating degenerative disorders associated with oxidative stress and neuronal dysfunction.

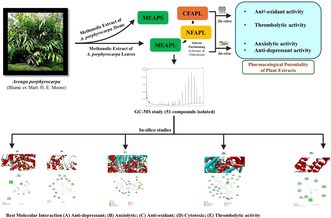

## 1 Introduction

Depression and anxiety are two of the most common psychiatric disorders worldwide (Horowitz and Vitkus 1986). These disorders cause irreversible damage, economic burden, and poor quality of life (Yong et al. 2009). The (WHO, 2001) report suggests that over 450 million people are universally experiencing mental health disorders (Fahad et al. 2021). The actual causes of anxiety and depression include environmental, biological, psychological and genetically induced factors. (Berton and Nestler 2006). Major depressive disorder (MDD) includes persistent depressive mood, loss of interest in activities, and significant cognitive impairment. It is the leading cause of global disability, affecting 300 million people and leading to 800,000 suicides yearly. (Jesulola et al. 2018; Martins and Brijesh 2018). The neurobiology of depression results from genetic and environmental factors, leading to cellular and molecular anomalies. The monoamine hypothesis, suggests that depression may result from decreased levels of serotonin (5‐hydroxytryptamine or 5‐HT), norepinephrine, and dopamine in the brain; most antidepressants target this mechanism. (Duman 2014; Lee et al. 2013). However, potential causes of depression include hypothalamic–pituitary–adrenal (HPA) axis dysfunctions, impaired neurogenesis, signaling pathway abnormalities, high inflammatory cytokines, and corticotrophin‐releasing factor dysregulations (Lopresti et al. 2012). While multiple antidepressants exist for depression, achieving complete relief remains challenging due to drawbacks like low response rates and high side effects. (Duman 2014; Inserra et al. 2019). Recent research shows the pivotal role of oxidative stress in disrupting the balance between harmful molecules and antioxidants, leading to depression. (Yanik et al. 2004; Bouayed 2010). Increased oxidative and nitrosative stress markers (e.g., nitric oxide (NO), malondialdehyde, and 8‐hydroxy‐2‐deoxyguanosine), along with reduced antioxidants (e.g., vitamin C, vitamin E, and coenzyme Q10), are linked to brain damage and may worsen depressive symptoms. (Bouayed 2010; Jahan et al. 2020; Škerget et al. 2005; Maia et al. 2019; Maes et al. 2011). Besides, many studies have linked this OS to various degenerative disorders such as cancer, osteoarthritis, aging, inflammatory disorders, cardiovascular disease, and osteoporosis. (Maes et al. 2011; Leyane et al. 2022).

Current research has been focused on safer compounds to treat various disorders, possibly developing a medicine effective against psychiatric illnesses and oxidative stress with a good safety record. Fortunately, the study of medicinal plants has made great advancements to explore possible safe and effective therapeutic candidates (Fahad et al. 2021; Moragrega and Ríos 2021). Medicinal plants are considered an asset of ancient medicines. (Qadir and Raja 2021). Moreover, medicinal herbs have been used for centuries for therapeutic, culinary, and aromatic purposes. Those are worth over $83 billion worldwide according to WHO, and are still used by 65%–80% of people in developing countries (Neves et al. 2009; Dièye et al. 2008).

*Arenga porphyrocarpa (Blume ex Mart. H. E. Moore)*, a tree from the Arecaceae family that is native to Indonesia and Malaysia, treats bladder problems, fever, loss of appetite, and is used as a diuretic. (Pillai et al. 2021). There are few scientific reports on this plant, especially regarding its potential for managing psychiatric conditions. Our study aims to evaluate its anxiolytic, anti‐depressant, antioxidant, and thrombolytic properties of the two organic soluble fractions of *A. porphyrocarpa*.

## 2 Materials and Method

### 2.1 Chemicals and Reagents

To conduct the investigation, various chemicals and reagents were sourced, including methanol, potassium ferricyanide, hydrochloric acid, aluminum chloride, sulfuric acid potassium acetate, Folin–Ciocalteu Reagent (FCR), and sodium carbonate, 1,1‐diphenyl‐2‐picrylhydrazyl (DPPH), sodium acetate, ferric chloride, quercetin, gallic acid and trichloroacetic acid (TCA), diclofenac sodium and diazepam and lyophilized streptokinase vial (1,500,000 IU) and vincristine sulfate (1 mg/vial). Square Pharmaceutical Ltd. and Beacon Pharmaceutical Ltd. provided specific substances. Absorbance was measured using an ultra‐violet‐vis spectrophotometer and most of the chemicals and reagents are analytical grade and the purity of reagents is 97%–99%.

### 2.2 Plant Collection and Extract Preparation

*A. Porphyrocarpa* plant was collected from Ruposhi Bangla Waterfall, Sitakund, Chattogram, Bangladesh. The sample was authenticated by Shaikh Bokhtear Uddin, Professor and Taxonomist, Department of Botany, University of Chittagong, Chittagong‐4331, Bangladesh. The leaves and stems were cleaned, air‐dried for 14 days at room temperature, and ground into coarse powder using a grinder (model number CK2686).

The plant extract was obtained using the cold extraction method, in which 10 g of plant materials was soaked with 100 mL of solvent (1:10 w/v) in amber glass bottles at room temperature and allowed to be treated for a few days (10–14 days) with random shaking and blending, followed by filtration through Whatmann No. 1 channel paper. The concentrated solution was then dried in a centrifugal evaporator at 40°C–50°C. The resulting deep green semi‐solid extracts from *A. porphyrocarpa* leaves and stems were kept in a refrigerator and used as methanol extract, yielding leaves (MEAPL) 7.6 g and stems (MEAPS) 4.2 g, respectively.

### 2.3 Solvent‐Solvent Partitioning

The Kupchan et al. (1973) formula underwent solvent‐solvent partitioning. (VanWagenen et al. 1993). After trituration, 5 g of leaves methanolic extract was mixed in 90% methanol. The n‐hexane fraction of *A. porphyrocarpa* (NFAPL) and chloroform fraction of *A. porphyrocarpa* (CFAPL) were evaporated for further analysis. NFAPL and CFAPL yielded 1.8 and 1.2 g each, stored for later investigation (Islam et al. 2020). The P&D committee (Pharm‐P&D17/08′‐19), Department of Pharmacy, International Islamic University Chittagong, Chittagong, Bangladesh, approved all study methodologies.

### 2.4 GC–MS Analysis of *A. porphyrocarpa* Leaves

We utilized a Shimadzu TQ 8040 mass spectrometer along with a GC‐17A gas chromatograph equipped with a DB‐1 coated capillary column (Rxi‐5 ms; 30 m length, 0.32 mm diameter, 0.25 μm film) to analyze a methanol extract of *A. Porphyrocarpa* leaves (MEAPL) through GC–MS. The GC oven had a specific temperature program, starting at 70°C and ramping to various temperatures followed by a hold period, while the detector and injector temperatures were set at 280°C and 230°C, respectively. The method involved a helium gas flow rate of 0.6 mL/min at constant pressure, and a 1 μL sample was analyzed in split mode for 50 min for compound identification based on peak area comparison with NIST and WILEY libraries in the GC–MS dataset.

### 2.5 Experimental Animals and Ethical Statement

Swiss Albino mice, both sexes, 22–26 g, were acquired from the Laboratory Animal Center of Jahangirnagar University, Bangladesh, kept in plastic cages (120 × 30 × 30 cm) at 25°C–28°C temperature, 55%–60% humidity with a 12‐h light and 12‐h dark cycle. Animal experiments were carried out following approval from the Institutional Animal Ethical Committee [Reference No: IIUC/PHARM‐AEC‐150/20–2019] of the Department of Pharmacy, International Islamic University Chittagong, Bangladesh (Zimmermann 1983). The report followed guidelines for narrating animal research and complied with “Animal Research: Reporting of In‐Vivo Experiments.” Animal care adhered to “Principles of Laboratory Animal Care” (NIH publication no. 85–23, revised 1985) and “National Animal Care Laws.” Mice were acclimated for 14 days and kept pain‐free. They were then euthanized using diethyl ether anesthesia.

### 2.6 Test of Acute Oral Toxicity

In the acute oral toxicity test following OECD Guidelines, Swiss albino mice were given several doses of all the plant extracts given orally daily (200, 400, 1000 and 2000 mg/kg, *n* = 6) after an 18‐h fast (Oecd, T 2002; Islam et al. 2025). The control group mice were given only vehicles (tween‐80 solution), and all the mice were then observed for allergic reactions like skin rash, itching, swelling, coma, diarrhea, salivation and mortality within 72 h.

### 2.7 Experimental Design

Experimental mice were divided into standard, control, and test groups, each consisting of six Swiss albino mice. Different extracts and fractions of MEAPL (methanolic extract of *A. porphyrocarpa* leaves), MEAPS (methanolic extract of *A. porphyrocarpa* stem), NFAPL (n‐hexane fraction of *A. porphyrocarpa* leaves) and CFAPL (chloroform fraction of *A. porphyrocarpa* leaves) were given to test groups at 200 and 400 mg/kg dosages orally, with the control group receiving a solution of 1% Tween‐80. Diazepam (1 mg/kg, b.w., i.p.) and imipramine (20 mg/kg) were used as standard groups for the elevated plus maze test (EPM), hole board test (HBT) and tail suspension test (TST) and forced swimming test (FST). Pre‐treatment with plant extracts and solvents occurred 30 min before, while diazepam was administered 15 min before the experiments took place.

### 2.8 *In‐Vivo* Anxiolytic Activity

#### 2.8.1 Elevated Plus Maze Test

The elevated plus maze (EPM) was used to assess plant extract's anxiolytic effects (Sarkar et al. 2024). The elevated plus maze (EPM) apparatus was constructed of wood and consisted of two opposing closed arms measuring 50 × 10 × 30 cm (length × width × height) and two opposing open arms measuring 50 × 10 cm (length × width), arranged in a plus‐shaped configuration. The maze was elevated 70 cm above the floor. Each mouse received the designated treatment before being placed at the center of the maze, facing one of the closed arms. An arm entry was defined as the mouse placing all four paws onto an arm. Mice movements were tracked as they explored the arms, recording time and entries in open and closed arms. During a 4‐min observation period, the time spent in the open and closed arms was recorded. All experiments were performed in a sound proof environment, and the maze was thoroughly cleaned with 70% ethanol between trials. Percentages of time spent and entries in each arm were documented as results.

#### 2.8.2 Hole Board Test

The anxiety‐provoking behavior of mice was assessed using the hole‐board test (HBT) (Sonavane et al. 2002). The test involved placing Swiss albino mice in a board with 16 holes (diameter 3 cm) raised 25 cm above the floor. Different doses were administered to each group of mice. After this, the mice were allowed to explore freely for 5 min, and the number that dipped their heads through the holes was recorded.

### 2.9 *In‐Vivo* Antidepressant Activity

#### 2.9.1 Tail Suspension Test

The Steru et al. method was used in the tail suspension test (TST) to explore the antidepressant potential of crude extracts (Steru et al. 1985). Different doses were given to separate groups of mice, followed by suspending each mouse by the tail with a clamp (1 cm from the end) in a box where the head was 5 cm from the bottom for 6 min. The test took place in a dark room, and the duration of immobility was measured in the final 4 min.

#### 2.9.2 Forced Swimming Test

Porsolt's theory was utilized in the forced swimming test (FST) to assess anti‐depressant activity in mice (Porsolt et al. 1977). Different doses were given to each mouse group. Mice were induced to swim for 6 min in water up to a height of 10 cm in cylinders (height: 20 cm; diameter: 14 cm). After drying, mice swam for 6 more minutes the next day. Immobility was observed during the last 4 min of the test.

### 2.10 *In‐Vitro* Antioxidant Test

#### 2.10.1 DPPH Radical Scavenging Activity

Barca et al. developed a technique to measure extract scavenging of 1,1‐diphenyl‐2‐picrylhydrazyl (DPPH) (Braca et al. 2001). Various concentrations of crude extracts (31.25 to 500 μg/mL) were mixed with 3 mL of DPPH solution (0.004%). Methanol with DPPH solution acted as a negative control. The mixtures were incubated at room temperature in the dark for 30 min and then their absorbance at 517 nm was measured using a UV spectrophotometer against a blank methanol solution. The standardized ingredient was ascorbic acid.

$$\%\text{Scavenging Activity}=\left(\mathrm{Ac}-\mathrm{As}\right)/\mathrm{Ac}\times 100$$

Here, Ac = The absorbance of control and As = the absorbance of the sample.

#### 2.10.2 Reducing Power Assay

Oyaizu method has been employed to accurately assess how much power of extracts was reduced substantially (Islam et al. 2022). Potassium ferricyanide (1% w/v) was added to 2.5 mL of phosphate buffer (0.2 M, pH 6.6) and approximately 1 mL of diluted concentration, ranging from 31.25 to 500 μg/mL. The solution was incubated at 50°C for 20 min to finish the procedure. 2.5 mL of 10% trichloroacetic acid was added after incubation, and the mixture was centrifuged for 10 min at 3000 RPM. The supernatant solution was then discarded and 0.5 mL of ferric chloride (0.1 w/v) was therefore incorporated into the mixture, whereas 2.5 mL of distilled water was gently introduced. Consequently, a UV spectrophotometer was utilized to investigate the absorbance at 700 nm. Ascorbic acid and phosphate buffer, respectively, were used as standards and controls.

#### 2.10.3 Total Phenolic and Flavonoid Content (Quantitative Phytochemicals)

The total phenolic content of the plant extracts was calculated using the Folin–Ciocalteu technique (Singleton et al. 1999), and expressed as mg of gallic acid equivalents. A 500 μg/mL extract was mixed with 2.5 mL of 20% sodium carbonate and 2.5 mL of Folin–Ciocalteu reagent (diluted 1:10), then diluted to 10 mL with distilled water and incubated at 25°C for 20 min. Absorbance was measured at 760 nm, and total phenolic content (TPC) was calculated from a gallic acid calibration curve and expressed as mg gallic acid equivalents.

On the other hand, the total flavonoid content (TFC) of the plant extracts was measured by using aluminum chloride (AlCL_3_) and referred to as mg of quercetin equivalent (Chang et al. 2002). A 500 μg/mL extract was mixed with 100 μL of 10% aluminum chloride, 1.5 mL methanol, 100 μL of 1 M potassium acetate, and 2.8 mL distilled water, then incubated at 37°C for 30 min. Absorbance was measured at 415 nm using a UV spectrophotometer, and total flavonoid content was calculated from a quercetin calibration curve and expressed as mg quercetin equivalents per gram.

### 2.11 In Vitro Thrombolytic Activity Assay

Prasad et al. investigated the thrombolytic potential of plant extracts (Prasad et al. 2006). A stock solution was prepared by diluting 5 mL of sterile distilled water with a vial of lyophilized streptokinase (1,500,000 I.U.). Blood samples (5 mL each) were collected from six volunteers, all of whom had not used anticoagulant medications recently. The blood was divided into six pre‐weighed sterile microcentrifuge tubes (0.5 mL each) and incubated at 37°C for 45 min. Serum was carefully withdrawn to avoid disrupting clot formation, and the clots were reweighed. Each tube acquired a portion of plant extract corresponding to 100 μL (10 mg/mL). Additionally, 100 μL of saline (negative control) and streptokinase (positive control) were added to the respective tubes. The tubes were incubated again for 1.5 h at 37°C. After incubation, the released fluid was cleaned, and the tubes were weighed once more to determine the weight difference due to clot disruption. Through the formula, the proportion of clot lysis was determined.

$$\begin{array}{c} \left(\%\right)\;\text{of clot lysis}\\ =\left(\text{The weight of clot after removing the fluid}/\text{The weight of clot}\right)\times 100 \end{array}$$

The ethical guidelines and declaration of Helsinki outlined in 1964 were followed in conducting this experiment involving humans. This is conducted by using authorized norms.

### 2.12 Statistical Analysis

All findings were analyzed with GraphPad Prism 7.0, utilizing ANOVA followed by Dunnett's test. A significant difference between groups was indicated by a *p*‐value below 0.05. Results are presented as the mean ± SEM (standard error of the mean).

### 2.13 In Silico Studies and Computational Approaches

#### 2.13.1 Ligand and Enzyme Preparation

A total of 15 significant compounds from MEAPL were selected by GC–MS analysis, PASS prediction, and extensive literature queries. The 3D structure of selected compounds was retrieved from the PubChem database (https://pubchem.ncbi.nlm.nih.gov). The Schrödinger suite‐Maestro (v11.1) was utilized to embed the molecular structures of these isolates, and the LigPrep method (forced field OPLS 2005) was employed to minimize energy. Three‐dimensional crystal enzyme structures have been obtained in PDB format from the Protein Databank for structural bioinformatics (Available from: https://www.rcsb.org/) (Mukit 2024; Shelley et al. 2007). For anti‐oxidant activity, human erythrocyte catalase (PDB ID: 1DGH) (Hossen et al. 2022); for antidepressant activity, (PDB ID: 6VRH) (Khan et al. 2020); for anxiolytic activity, bromodomain of human BRD4 in complex with midazolam (PDB ID: 3U5K) (Jyoti et al. 2020); for thrombolytic activity, (PDB ID: 3PP0) (Shovo et al. 2021); for cytotoxic activity (PDB ID: 1A5H) (Mahnashi et al. 2021) were adopted for molecular docking from previously published data.

#### 2.13.2 Grid Generation and Extra‐Precision (XP) Molecular Docking Analysis

As previously noted (Khan et al. 2020), Schrödinger Maestro (v11.1) was used to conduct docking investigations between receptors and ligands. Subsequently, scores were collected utilizing a variety of enzymes and ligand geometries were compared with the geometry of the reference (standard drug) ligand, without changing the default docking setups in XP mode. Each docking was done three times to guarantee correctness. The ultimate score, represented as the glide %, was calculated using energy‐minimization procedures. After sorting the ligands using the Glide score, the least binding score for each ligand was noted. The glide score and glide energy were the two primary measures used to assess the docking result. Eventually, the docked components were photographed and stored for the display of the docking picture in the .sdf format. The grid dimensions were set to [14 × 14 × 14 Å^3^], which was sufficient to cover the binding pocket and allow optimal ligand flexibility during docking.

#### 2.13.3 In Silico ADME and Drug‐Likeness Analysis

Lipinski's rule of five (RO5) was adopted to determine whether a compound has drug‐like characteristics. A chemical may be characterized as drug‐like if it satisfies the primary set of requirements, which include molecular weight, H‐bond recipient, H‐bond donor, TSPA, and Log *p*‐value within certain ranges. However, it cannot circumvent more than one of Lipinski's rules. A web‐based program called Swissadme was additionally employed to examine the ADME characteristics of the selected substances.

## 3 Results

### 3.1 GC–MS Analysis

**FIGURE 1:**
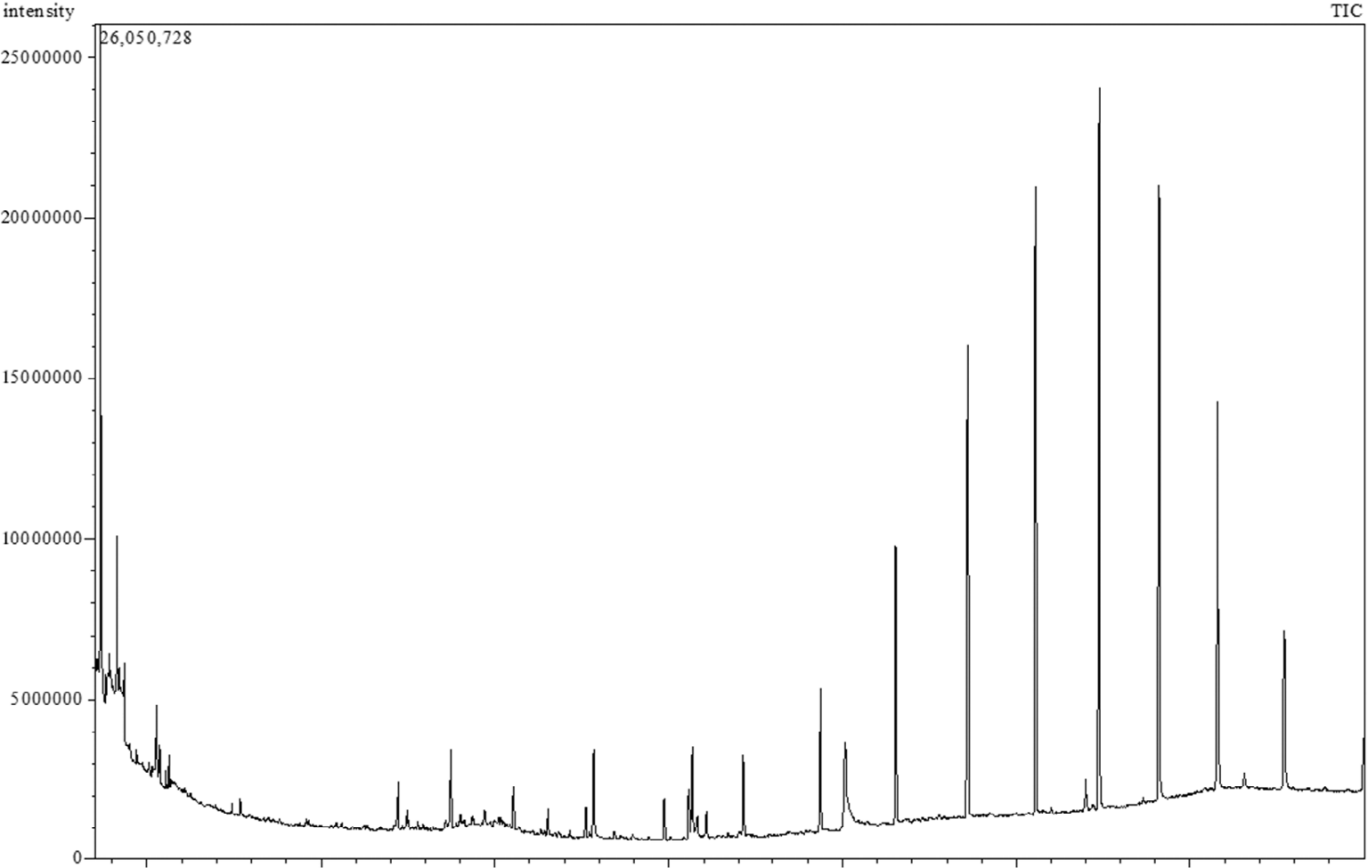
Total ionic chromatogram (TIC) of the methanol extract of *A. porphyrocarpa* leaves (MEAPL) using gas chromatography mass spectrometry (GC–MS).

The GC–MS analysis (Figure 1) of MEAPL identified 51 bioactive compounds with diverse retention times and concentration ranges, as shown in Table S1. Several of the major metabolites detected in our GC–MS analysis (e.g., neophytadiene, phytol, squalene, hexadecanoic acid methyl ester, linoleic acid methyl ester, oleic acid methyl ester, and Vitamin E) are well known for their antioxidant, antimicrobial, anti‐inflammatory, cytoprotective and neuropharmacological roles, as documented in earlier studies.

### 3.2 Acute Oral Toxicity Test (In Vivo)

Acute toxicity evaluation of all extract fractions at doses of 200, 400, 1000, and 2000 mg/kg body weight showed no visible signs of toxicity or mortality over 3 days, indicating that the lethality dose (LD_50_) of the extract is greater than 2000 mg/kg.

### 3.3 Anxiolytic Activity

#### 3.3.1 Elevated Plus Maze Test (EPM)

EPM investigation revealed that all plant extracts exhibited dose‐dependent effects at 200 and 400 mg/kg, particularly in terms of time spent and frequency of entries into the open arm (Table 1). Notably, the highest anxiolytic activity was observed with a 400 mg/kg dose of MEAPL, NFAPL, and CFAPL, highlighting their potential as anxiolytic agents. Among these, CFAPL demonstrated the greatest efficacy, significantly increasing the number of entries (58.28% ± 0.90%) and time spent (55.50% ± 1.64%) in the open arm at this dose (*p* < 0.001). Additionally, administration of the standard drug diazepam at 1 mg/kg resulted in 78.45% ± 1.26% and 84.26% ± 1.53% for time spent and entries into open arms, respectively.

**TABLE 1:** The anxiolytic behavior of *A. porphyrocarpa* fractions and diazepam (standard) in an elevated plus maze test (EPM) in mice.

Treatment dose (mg/kg)	Entry into open arm (%)	Time spent in open arm (%)
Control	34.16 ± 1.47	29.60 ± 1.30
Standard	84.26 ± 1.53^a^	78.45 ± 1.26^a^
MEAPL‐200	44.82 ± 1.42^c^	37.17 ± 1.83^ns^
MEAPL‐400	53.29 ± 1.14^a^	56.41 ± 2.23^a^
MEAPS‐200	34.42 ± 1.74^ns^	32.35 ± 1.87^ns^
MEAPS‐400	45.14 ± 1.69^b^	41.37 ± 1.92^d^
NFAPL‐200	36.42 ± 2.07^ns^	43.35 ± 1.01^c^
NFAPL‐400	49.98 ± 0.85^a^	55.18 ± 1.38^a^
CFAPL‐200	45.16 ± 1.75^c^	48.15 ± 1.85^a^
CFAPL‐400	58.28 ± 0.90^a^	55.50 ± 1.64^a^

*Note:* The results are expressed as the mean ± SEM, whereas ^d^ *p* < 0.05, ^c^ *p* < 0.01, ^b^ *p* < 0.001, and ^a^ *p* < 0.0001 are considered statistically significant. The statistical analysis was followed by a one‐way analysis of variance (Dunnett's test), and the results were compared to the negative control (1% Tween‐80) using GraphPad Prism version 7.0.

Abbreviations: CFAPL, chloroform fraction of *A. porphyrocarpa* leaves; MEAPL, methanolic extract of *A. porphyrocarpa* leaves; MEAPS, methanolic extract of *A. porphyrocarpa* stems; NFAPL, n‐hexane fraction of *A. porphyrocarpa* leaves.

#### 3.3.2 Hole Board Test (HBT)

In this test, mice receiving 200 and 400 mg/kg doses of *A. porphyrocarpa* demonstrated a dose‐dependent increase in hole poking behavior (Table 2). The 400 mg/kg dose significantly (*p* < 0.001) exhibited the most pronounced anxiolytic effects, with MEAPL at 65.16 ± 2.33 and CFAPL at 65.83 ± 1.45. Additionally, the standard drug showed a significant increase in head dipping frequency (89.66 ± 1.76) compared to the control group.

**TABLE 2:** The anxiolytic potentiality of *A. porphyrocarpa* fractions in a hole board test (HBT) in mice.

Treatment dose (mg/kg)	No. of head dipping
Control	27.16 ± 0.88
Standard	89.66 ± 1.76^a^
MEAPL‐200	47.33 ± 1.45^b^
MEAPL‐400	65.16 ± 2.33^a^
MEAPS‐200	40.50 ± 2.18^d^
MEAPS‐400	59.33 ± 1.85^b^
NFAPL‐200	34.56 ± 1.20^ns^
NFAPL‐400	51.83 ± 1.86^b^
CFAPL‐200	45.16 ± 1.88^a^
CFAPL‐400	65.83 ± 1.45^a^

*Note:* The results are expressed as the mean ± SEM, whereas ^d^ *p* < 0.05, ^c^ *p* < 0.01, ^b^ *p* < 0.001, and ^a^ *p* < 0.0001 are considered statistically significant. The statistical analysis was followed by a one‐way analysis of variance (Dunnett's test), and the results were compared to the negative control (1% Tween‐80) using GraphPad Prism version 7.0.

Abbreviations: CFAPL, chloroform fraction of *A. porphyrocarpa* leaves; MEAPL, methanolic extract of *A. porphyrocarpa* leaves; MEAPS, methanolic extract of *A. porphyrocarpa* stems; NFAPL, n‐hexane fraction of *A. porphyrocarpa* leaves.

### 3.4 Anti‐Depressant Activity

#### 3.4.1 Tail Suspension Test (TST)

**FIGURE 2:**
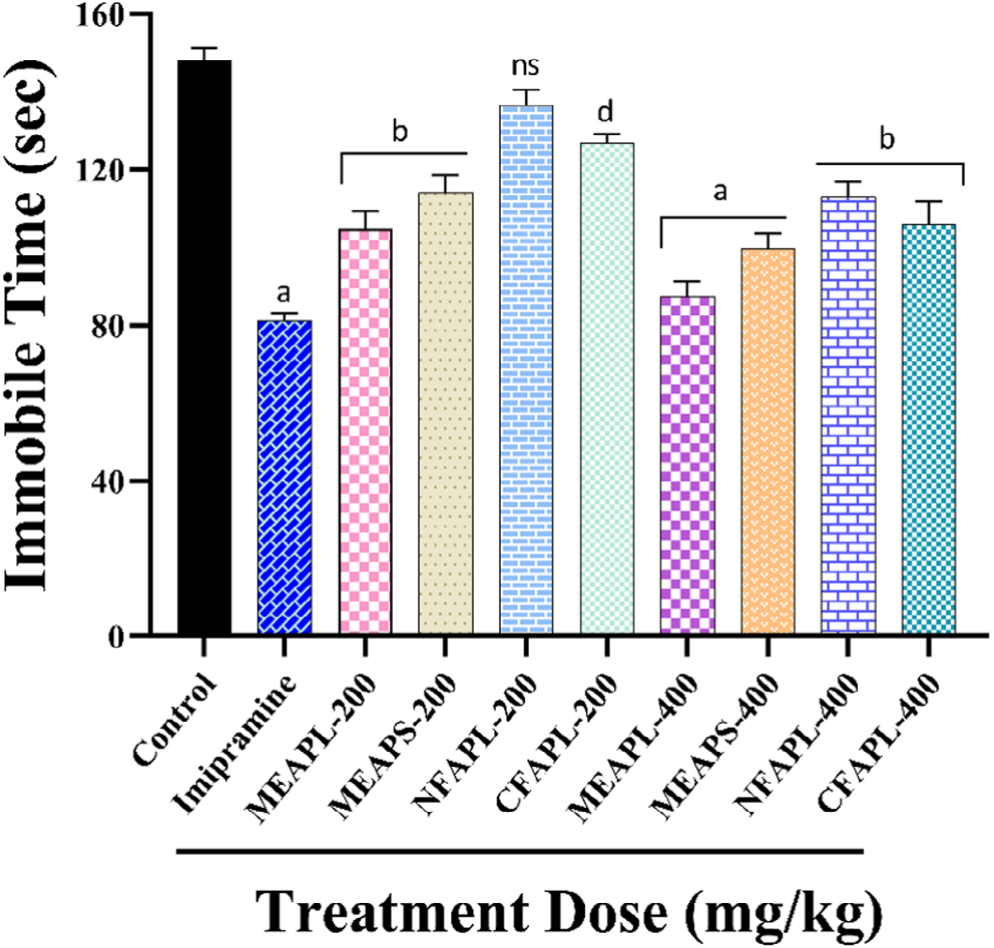
Antidepressant activity of *A. porphyrocarpa* on tail suspension tests (TST) in mice. The values are expressed as the mean ± SEM; one‐way analysis of variance (ANOVA) followed by Dunnett's test; whereas ^d^ *p* < 0.05, ^c^ *p* < 0.01, ^b^ *p* < 0.001, and ^a^ *p* < 0.0001 are considered statistically significant. compared to the negative control (1% Tween‐80) using GraphPad Prism version 7.0. CFAPL, Chloroform fraction of *A. porphyrocarpa* leaves; MEAPL, Methanolic extract of *A. porphyrocarpa* leaves; MEAPS, Methanolic extract of *A. porphyrocarpa* stems; NFAPL, N‐hexane fraction of *A. porphyrocarpa* leaves.

In the anti‐depression test using the tail suspension test (TST), all crude extract results are summarized in Figure 2. Both 200 and 400 mg/kg doses of the plant extracts resulted in a notable reduction in immobility time, demonstrating a dose‐dependent effect (*p* < 0.05). Among the extracts, MEAPL significantly (*p* < 0.001) exhibited the highest inhibition of immobility at 87.55 ± 2.16 s (40.8%) with a 400 mg/kg dose, closely resembling the reference drug Imipramine, which recorded 81.24 ± 1.02 s (45.06%) at a 10 mg/kg dose.

#### 3.4.2 Forced Swimming Test (FST)

**FIGURE 3:**
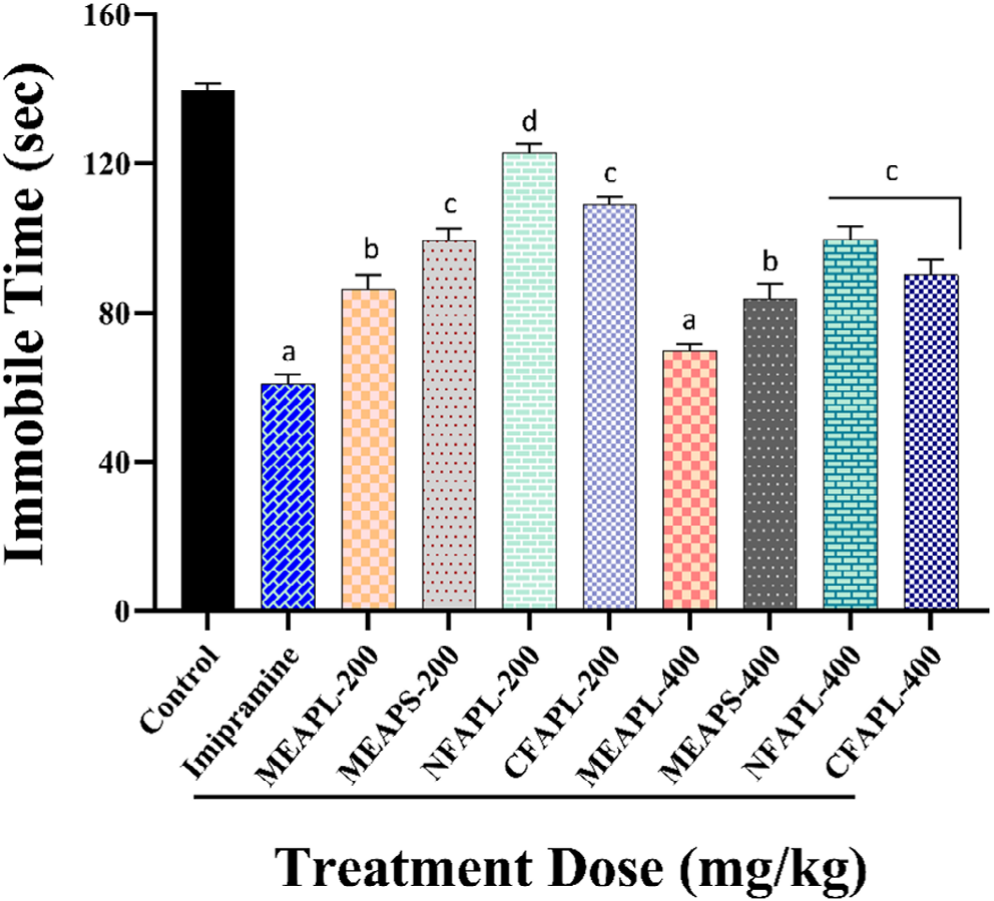
Investigation of the antidepressant activity of *A. porphyrocarpa* fractions in a forced swimming test (FST) in mice. The results are expressed as the mean ± SEM; one‐way analysis of variance (ANOVA) followed by Dunnett's test; whereas ^d^ *p* < 0.05, ^c^ *p* < 0.01, ^b^ *p* < 0.001, and ^a^ *p* < 0.0001 are considered statistically significant compared to the negative control (1% Tween‐80) using GraphPad Prism version 7.0. CFAPL, Chloroform fraction of *A. porphyrocarpa* leaves; MEAPL, Methanolic extract of *A. porphyrocarpa* leaves; MEAPS, Methanolic extract of *A. porphyrocarpa* stems; NFAPL, *N*‐hexane fraction of *A. porphyrocarpa* leaves.

In the FST investigation, control group mice exhibited behavioral despair, characterized by immobility. In contrast, mice treated with plant extracts (200 and 400 mg/kg) and the reference drug imipramine (10 mg/kg) displayed active behaviors, such as swimming and struggling, as shown in Figure 3. Increased doses of the plant extracts resulted in longer mobility times, suggesting that *A. porphyrocarpa* has antidepressant properties. Among the extracts, MEAPL at 400 mg/kg demonstrated the most significant (*p* < 0.001) effect, with a 69.92% ± 1.04% (49.89%) reduction in immobility. Additionally, imipramine reduced immobility time by 78%, recording a time of 60.99 ± 1.41 s.

### 3.5 Anti‐Oxidant Activity

#### 3.5.1 DPPH Radical Scavenging Assay

**FIGURE 4:**
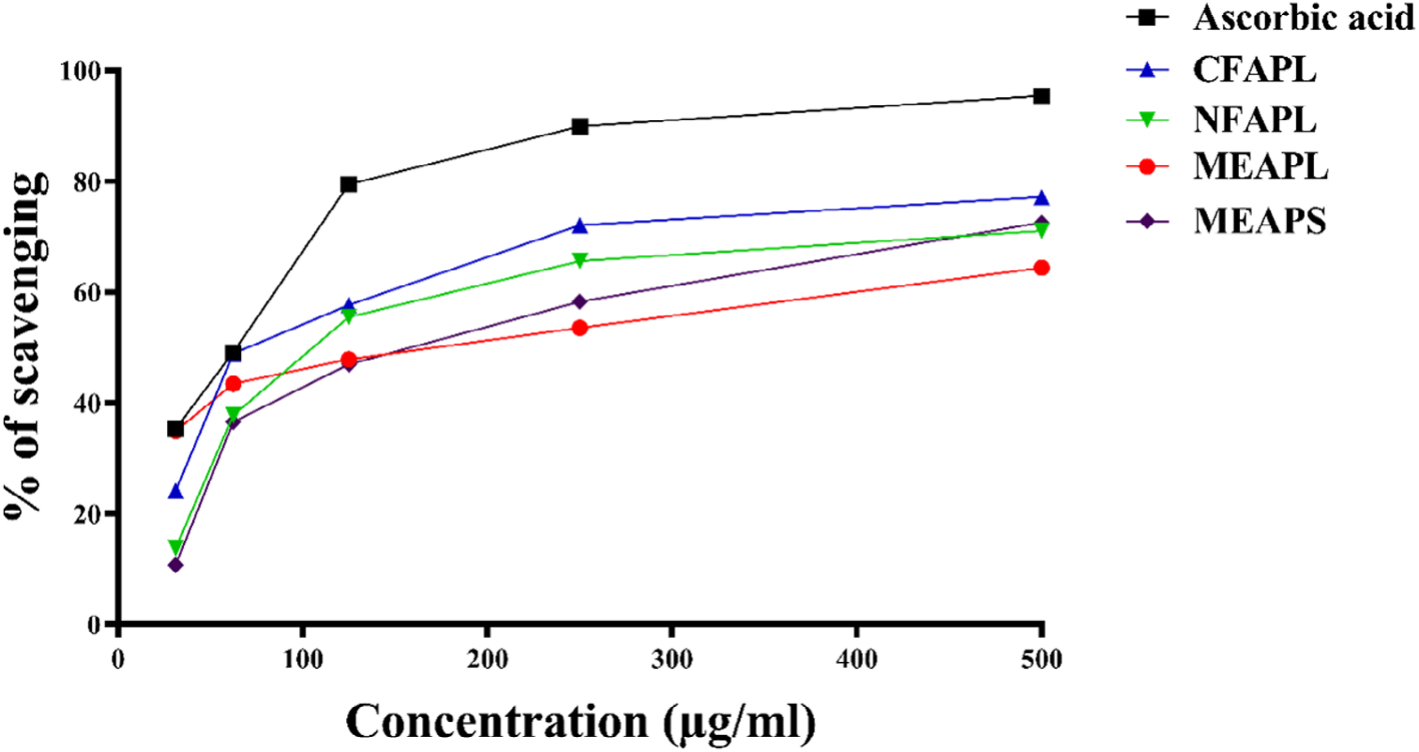
Percentage (%) of radical scavenging activity by the DPPH of *A. porphyrocarpa* fractions and reference standard drug ascorbic acid at different concentrations.

Figure 4 and Table 3 illustrate the antioxidant activity of *A. porphyrocarpa* fractions, with CFAPL showing a strong antioxidant potential (IC_50_ = 128.94 μg/mL) compared to the reference standard drug ascorbic acid. The plant extracts showed dose‐dependent antioxidant activity in the DPPH assay, with higher concentrations producing stronger free radical scavenging comparable to the standard drug, ascorbic acid. The order of DPPH scavenging activity is as follows: ascorbic acid > CFAPL > NFAPL > MEAPL > MEAPS, which indicates that CFAPL exhibits a notable antioxidant effect, reinforcing the potential of *A. porphyrocarpa* fractions in contributing to antioxidant defense mechanisms.

**TABLE 3:** IC_50_ values with regression equation for *A. porphyrocarpa* fractions with reference to ascorbic acid.

IC_50_ values (μg/mL) of radical scavenging		
Chemical/plant extract	IC_50_	Regression equation
Ascorbic acid	20.29	*Y* = 0.1144X + 47.68; *R* ^2^ = 0.6867
MEAPL	215.04	*Y* = 0.0555× + 38.065; *R* ^2^ = 0.9165
MEAPS	239.87	*Y* = 0.1083× + 24.021; *R* ^2^ = 0.7795
NFAPL	206.49	*Y* = 0.09935X + 29.47; *R* ^2^ = 0.6613
CFAPL	128.95	*Y* = 0.09217X + 38.11; *R* ^2^ = 0.6946

Abbreviations: CFAPL, chloroform fraction of *A. porphyrocarpa* leaves; MEAPL, methanolic extract of *A. porphyrocarpa* leaves; MEAPS, methanolic extract of *A. porphyrocarpa* stems; NFAPL, n‐hexane fraction of *A. porphyrocarpa* leaves.

#### 3.5.2 Reducing Power Assay

**FIGURE 5:**
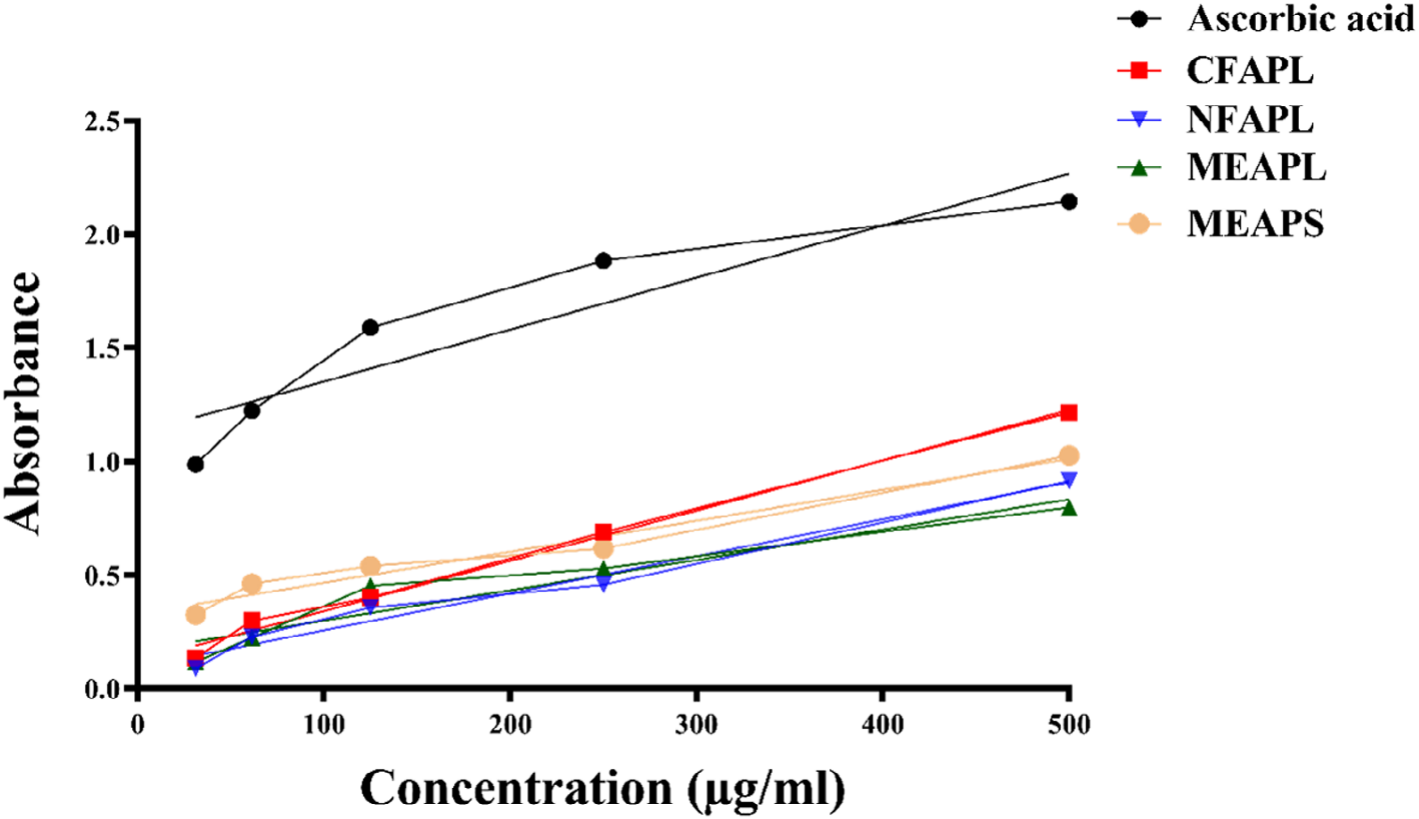
Power reducing assay of *A. porphyrocarpa* fractions and reference standard drug ascorbic acid at different concentrations.

Figure 5 illustrates the dose–response curve for the reducing power of plant extracts (31.25–500 μg/mL), showing that higher sample concentrations lead to increased reducing power. Among the extracts, CFAPL demonstrated the highest absorbance and a consistent increase with concentration, indicating robust antioxidant activity. The reducing power potential ranked as follows: Ascorbic acid > CFAPL > MEAPS > NFAPL > MEAPL.

### 3.6 Total Phenolic and Flavonoid Content (Quantitative Phytochemicals)

The total phenolic and flavonoid content of *A. porphyrocarpa* fractions (1000 μg/mL) is detailed in Table 4. MEAPL exhibited the highest phenolic content at 149.95 mg GAE/g of dried extract, followed by MEAPS with 117.45 mg GAE/g. For flavonoid content, CFAPL found the highest flavonoid content (193.78 mg QE/g), while MEAPL had 111.84 mg QE/g. Regression analyses yielded equations for total phenolic (*y* = 0.0004× + 0.1782, *R* ^2^ = 0.9759) and flavonoid (*y* = 0.0006× − 0.0017, *R* ^2^ = 0.9939) contents across all extracts.

**TABLE 4:** Total phenol and flavonoid contents (quantitative phytochemicals) of *A. porphyrocarpa* fractions.

Tested extracts	Total phenol content (mg GAE/g dried extract)	Total flavonoid content (mg QE/g dried extract)
MEAPL	117.45 ± 1.95	85.78 ± 1.58
MEAPS	149.95 ± 1.29	111.84 ± 1.97
NFAPL	94.20 ± 1.15	90.05 ± 1.22
CFAPL	78.11 ± 1.65	193.78 ± 1.92

Abbreviations: CFAPL, chloroform fraction of *A. porphyrocarpa* leaves; MEAPL, methanolic extract of *A. porphyrocarpa* leaves; MEAPS, methanolic extract of *A. porphyrocarpa* stems; NFAPL, n‐hexane fraction of *A. porphyrocarpa* leaves.

### 3.7 Thrombolytic Activity

Table 5 highlights the thrombolytic potential of *A. porphyrocarpa*. Compared to the negative control (water) at 4.31%, CFAPL exhibited the highest significant clot lysis percentage at 35.29% (*p* < 0.001) among the plant extracts. In contrast, the reference standard, streptokinase, showed a significantly higher clot lysis rate of 75.05% (*p* < 0.0001). The hierarchy of clot lysis efficacy is as follows: streptokinase > CFAPL > MEAPL > NFAPL > MEAPL > water, demonstrating the varying effectiveness of different treatments.

**TABLE 5:** Thrombolytic activity of *A. porphyrocarpa* fractions and streptokinase (standard).

Chemical/plant extract	Percentage (%) of clot lysis
Negative control (water)	4.31 ± 0.30
Positive control (streptokinase)	75.05 ± 0.15***
MEAPL	20.36 ± 2.58*
MEAPS	25.57 ± 1.75*
NFAPL	22.34 ± 1.40*
CFAPL	35.29 ± 1.25**

*Note:* Values are expressed as the mean ± SEM; one‐way analysis of variance (ANOVA) followed by Dunnett's test; whereas *p < 0.05, **p < 0.01, and ***p < 0.001 are considered statistically significant. compared to the negative control (1% Tween‐80) using GraphPad Prism version 7.0.

Abbreviations: CFAPL, chloroform fraction of *A. porphyrocarpa* leaves; MEAPL, methanolic extract of *A. porphyrocarpa* leaves; MEAPS, methanolic extract of *A. porphyrocarpa* stems; NFAPL, n‐hexane fraction of *A. porphyrocarpa* leaves.

### 3.8 In Silico Molecular Docking Analysis

Out of the 51 molecules identified predominantly via GC–MS screening, a captivating selection of 15 major molecules was chosen for molecular docking analysis, carefully curated based on their remarkable concentrations and retention times. Molecular docking scores of selected compounds with specific proteins are displayed in Table S2.

#### 3.8.1 Molecular Docking Analysis of Neuropharmacological Activity

**FIGURE 6:**
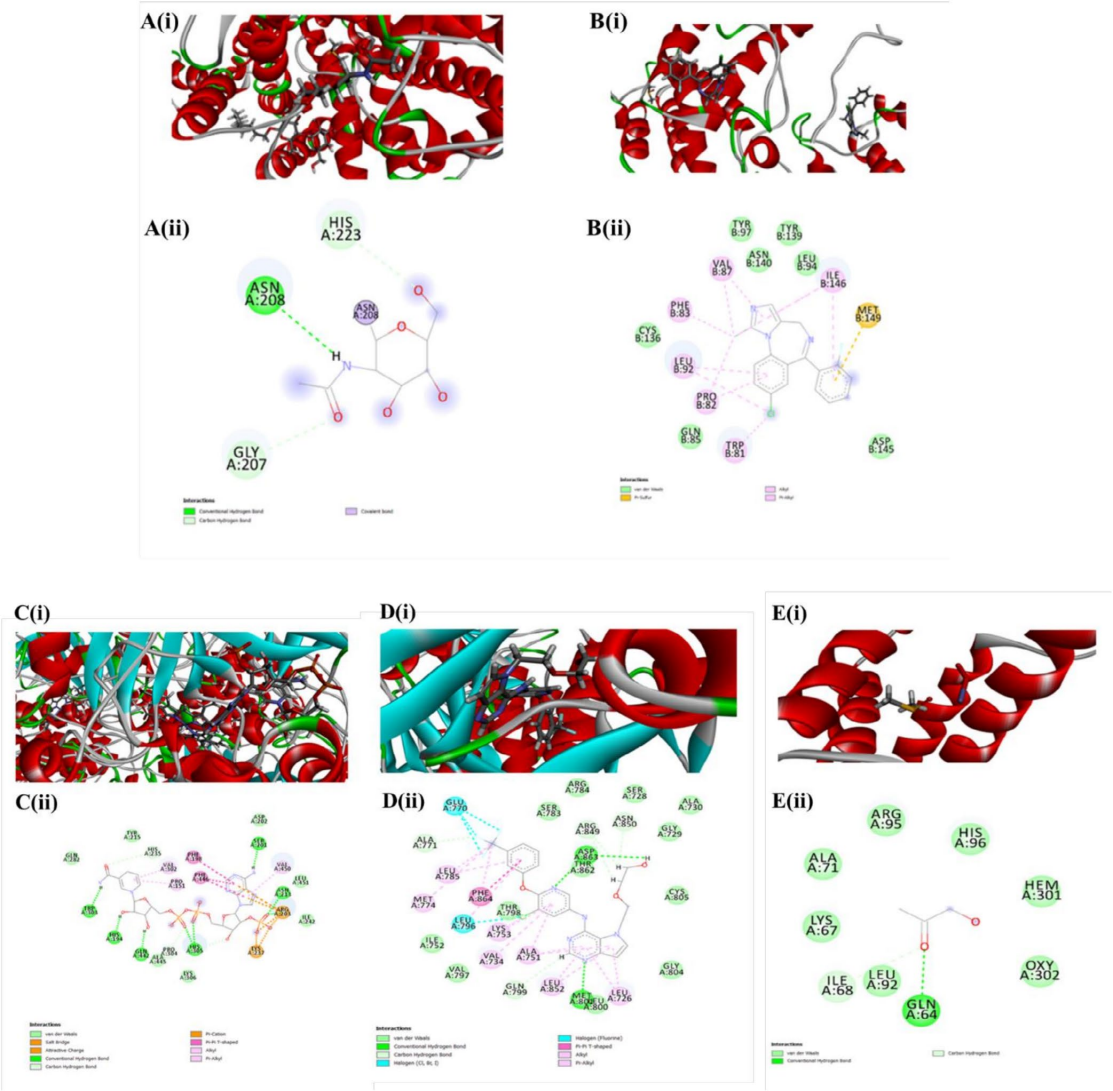
Molecular interaction of the best‐ranked poses for several target proteins (A–E, i) 3D image of 2‐cyclobutene‐1‐carboxamide with 6VRH for antidepressant activity; (A–E, ii) 2D image of 2‐cyclobutene‐1‐carboxamide with 6VRH for antidepressant activity; (B, i) 3D image of isobutyric acid, 2,2,2‐trichloroethyl ester with 3U5K for anxiolytic activity; (B, ii) 2D image of isobutyric acid, 2,2,2‐trichloroethyl ester with 3U5K for anxiolytic activity; (C, i) 3D image of 2‐pyridinecarboxylic acid with 1DGH for antioxidant potential; (C, ii) 2D image of 2‐pyridinecarboxylic acid with 1DGH for antioxidant potential; (D, i) 3D image of propionic acid, 3‐(isobutylthio)‐ with 3PP0 for cytotoxic activity; (D, ii) 2D image of propionic acid, 3‐(isobutylthio)‐ with 3PP0 for cytotoxic activity; (E, i) 3D image of 2‐propanone, 1‐hydroxy‐ with 1A5H for thrombolytic activity; (E, ii) 2D image of 2‐propanone, 1‐hydroxy‐ with 1A5H for thrombolytic activity.

Fifteen foremost molecules among those 51 molecules identified primarily by GC–MS screening and standard drugs (Fluoxetine HCl and Diazepam) were docked against various neuroprotective receptors, specifically for antidepressant activity (PDB ID: 6VRH); for anxiolytic activity (PDB ID: 3U5K); in this phase of investigation. From docking result analysis, 2‐Cyclobutene‐1‐carboxamide manifested the highest binding affinity values (−3.781 Kcal/mol^−1^) with 6VRH (Figure 6A). The strong H‐bond to Asn208 suggests the carboxamide is acting as a key recognition element—it likely mimics a natural hydrogen‐bonding motif used by endogenous ligands or substrates of 6VRH. This directional contact will improve specificity and contribute substantially to binding affinity. The presence of multiple weaker backbone/side‐chain contacts (Gly207, His223) indicates the ligand is snugly accommodated in the cavity rather than loosely surface‐bound. That compact fit increases the probability the ligand can modulate the target site relevant to antidepressant activity. No covalent modification is evident (only non‐covalent contacts), so the predicted mechanism is reversible binding.

The standard drug, fluoxetine exhibits (−9.863 Kcal/mol^−1^). Against the anxiolytic receptor 3U5K, isobutyric acid, 2,2,2‐trichloroethyl ester exhibits a docking score of (−5.333 Kcal/mol^−1^); compared to the standard diazepam exhibiting a score of −6.851 Kcal/mol^−1^ with 3U5K. (Figure 6B). The ligand is placed in a largely hydrophobic/aromatic pocket and shows many alkyl and π–alkyl interactions with residues such as Trp81, Pro82, Phe83, Leu92, Leu94, Val87, Tyr97, Tyr139, Ile146, and Met149 (pink dashed lines on the 2D map indicate π/alkyl contacts). A π–sulfur style interaction (yellow dashed) to Met149 is annotated, indicating the trichloroethyl/aryl portion is close enough to experience soft polarizable contacts with the methionine sulfur. There is numerous van der Waals contacts distributed across the pocket; no single strong conventional hydrogen bond is clearly dominant in the 2D figure.

#### 3.8.2 Molecular Docking Analysis of Antioxidant Activity

In terms of anti‐oxidant analysis, all the 15 selected molecules were docked with distinct receptors associated with antioxidant capacity, namely human erythrocyte catalase (PDB ID: 1DGH). In this analysis, 2‐Pyridinecarboxylic acid manifested the significantly highest docking value (−7.546 Kcal/mol^−1^) whereas standard drug ascorbic acid shows (−6.928 Kcal/mol^−1^) (Figure 6C). The 3D and 2D molecular interaction diagrams of 2‐Pyridinecarboxylic acid with the protein 1DGH reveal a strong and stable binding affinity, indicating promising antioxidant potential. The compound exhibits multiple interactions with key amino acid residues in the binding pocket of 1DGH, including conventional hydrogen bonds with residues such as PRO A:304 and ALA A:306, contributing to stability. Pi interactions (Pi‐Pi T‐shaped and Pi‐alkyl) with aromatic residues like PHE A:198 and VAL A:450 further enhance binding through hydrophobic stacking. Additionally, van der Waals interactions with residues such as TYR A:326, GLN A:282, and ILE A:322 provide complementary stabilization. The presence of pi‐cation interactions and salt bridges also suggests electrostatic attraction within the active site.

#### 3.8.3 Molecular Docking Analysis of Cytotoxic Activity

The cytotoxic potential has been studied by utilizing molecular docking interactions with the Kinase Domain of HER2 Protein (PDB ID:3PP0), and shown in Figure 6D. Among 15 molecules selected for the study, Propionic acid, 3‐(isobutylthio)‐ demonstrated the highest molecular binding affinity (−8.825 Kcal/mol^−1^) than the standard Vincristine Sulphate (−1.094 Kcal/mol^−1^). The predicted complex shows a convincing binding mode: a directional H‐bond to Asp863 anchors the ligand while halogen, π and extensive hydrophobic contacts (Leu/Val/Met/Phe cluster) supply strong van der Waals stabilization. This mixed polar–hydrophobic recognition supports the ligand's potential to bind tightly to 3PP0 and produce a functional effect.

#### 3.8.4 Molecular Docking Analysis of Thrombolytic Activity

The thrombolytic qualities of 15 selected molecules were assessed via molecular docking with a human tissue‐type plasminogen activator (PDB ID: 1A5H), and shown in Figure 6E. A correlation emerged between the aforementioned molecules' structural features, and biological properties toward specific receptors. Here, 2‐Propanone, 1‐hydroxy‐ exhibits the highest binding score (−4.903 Kcal/mol^−1^) among specified molecules with 1A5H compared to the standard drug streptokinase which has a docking score of (−5.535 Kcal/mol^−1^). Molecular docking of 2‐Propanone, 1‐hydroxy‐ with 1A5H (human fibrinogen fragment, relevant in thrombolytic activity) revealed a stable binding pose within the active site of the protein. The 3D binding conformation shows that the ligand is well accommodated in a hydrophobic pocket, surrounded primarily by helical regions, indicating a favorable structural environment for interaction.

The 2D interaction diagram highlights that Gln64 plays a crucial role in stabilizing the ligand through a conventional hydrogen bond, which enhances the binding affinity and specificity of the compound toward the protein. In addition, several residues, including Lys67, Ile68, Ala71, Leu92, Arg95, His96, and the heme group (HEM301), participate in van der Waals interactions, further stabilizing the ligand within the binding cavity. These non‐covalent interactions are critical, as they provide additional stabilization while maintaining flexibility, which is important for biological activity.

#### 3.8.5 In Silico ADME/T and Drug‐Likeness Analysis

The appropriateness of the compounds and their potential for use as therapeutic agents was examined in the current study. Due to the previously described rationale, numerous variables were considered: the molecular weight in grams (< 500 g/mol), the number of hydrogen bond donors (≤ 5), acceptors (≤ 10), logP (< 5), and molar refractivity (40–130) of the derivative molecules. As demonstrated in Table S3, it can be predicted that most of the derivative molecules considered have the potential to be explored as a potential drug candidate.

## 4 Discussion

Medicinal plants have historically served as a crucial source of medicine, as noted in both the Vedas and the Holy Bible, which highlight their use in herbal remedies and healthcare (Shakya 2016). These plants have long been utilized for food production, health treatment, and disease prevention, including epidemics (Sofowora et al. 2013). Compounds derived from these plants exhibit diverse biological activities, including anxiolytic, antioxidant, thrombolytic, and neurodegenerative properties. Consequently, a functional investigation was conducted to explore these effects further (Hamburger and Hostettmann 1991). Some phytochemicals may have mutagenic or genotoxic characteristics, despite the fact that many of them have wide biological and pharmacological functions. Therefore, in our experiment, cytotoxicity assays were used to provide an initial safety profile of the extracts, ensuring that the tested doses are not generally toxic. It complements acute toxicity observations in vivo, where behavioral monitoring with controls confirmed the absence of toxicity. These combined safety evaluations guided dose selection for further pharmacological studies and the prioritization of extracts for further analysis. In future research, it is suggested to conduct further cytotoxic tests (e.g., CCK‐8 or MTT assay) for further clarification.

The EPM is a widely recognized assessment that influences the anxiolytic drugs on the type A (GABAA) gamma‐aminobutyric acid benzodiazepine complex (Emon et al. 2020). A significant reduction in open arm exploration indicates an anxiolytic effect, characterized by increased time spent and entries in the open arm (Barua et al. 2012). In our study, all fractions consistently affected the frequency and duration of open arm exploration at doses of 200 and 400 mg/kg. Notably, at the 400 mg/kg dose, CFAPL demonstrated superior anxiolytic potential compared to other extracts, resulting in a 58.28% ± 0.90% increase in entries and a 55.50% ± 1.64% increase in time spent in the open arms. These behavioral changes align with anxiolytic activity. Greater hole‐poking behavior suggests heightened anxiolytic effects, while hesitation to approach the hole is interpreted as anxiety (Sillaber et al. 2008). In our investigation, administration of the plant extract at both 200 and 400 mg/kg doses significantly enhanced anxiogenic behavior in mice, with the highest activity observed at 400 mg/kg. These results may be attributed to the constituents of the extract influencing GABA‐t (gamma‐aminobutyric acid transaminase) activity, potentially reducing anxiety levels by elevating brain GABA concentrations (Emon et al. 2020).

Research has shown that the neuropharmacological phenomenon known as the “halo effect” can lead to a reduction in depression following effective anxiety treatment (Adnan, Chy, Kamal, et al. 2020). Depression, a mental illness characterized by mood disturbances, can manifest through symptoms such as sadness, loss, and anger that disrupt daily activities (Kumar et al. 2012). The current study investigated the antidepressant effects of A. porphyrocarpa fractions using forced swimming and tail suspension tests, both designed to induce a depressive state in mice. Results indicated that shorter immobility times correlate with greater antidepressant activity (Acerson et al. 2013). In the screening of TST, notably, a dose of 400 mg/kg of MEAPL exhibited a more significant reduction in immobility (40.8%) compared to the reference drug Imipramine (45.06%) at 10 mg/kg across various extract doses. In the forced swimming test, all doses of the plant extracts decreased immobility time in a dose‐dependent manner, suggesting their potential antidepressant properties. The highest immobility percentage was observed with MEAPL (49.89%), while Imipramine recorded 78%. Overall, MEAPL could be the best option to identify a lead compound for the anti‐depressant effect, and potentially linked to its total phenol and flavonoid content, as suggested by several prior studies (Guan and Liu 2016).

Nitric oxide radicals (*NO) and oxygen radicals (O2–1) are generated by inflammatory cells under pathogenic conditions, leading to the formation of the potent oxidizing agent peroxy‐nitrite anion (ONOO‐1). This anion diffuses into nitro‐sonium cations (NO+1) and nitroxyl anions (NO‐1) through mechanisms such as DNA fragmentation and lipid peroxidation, resulting in oxidative stress (OS) and various physiological dysfunctions. (Cardozo‐Pelaez et al. 1999; Dinh et al. 2014). Research indicates that cellular OS in the brain can disrupt normal neuronal processes, brain activity, and neurotransmission, linking OS to neuropsychiatric disorders. Antioxidant therapy may alleviate abnormal neural activities and OS by inhibiting reactive oxygen species (ROS) formation and modulating redox‐related signaling pathways. Natural antioxidants can be found in abundance in plants, and some phytochemicals also have antioxidant activities; whereas their main function is to guard against oxidative stress caused by free radicals (Adnan, Chy, Kama, et al. 2020; Rammal et al. 2008). Polyphenolic compounds, particularly flavonoids and phenolic acids, are of great interest due to their strong antioxidant properties found in fruits, vegetables, cereals, and beverages (Arts and Hollman 2005). Studies have shown that gallic acid and quercetin can reduce oxidative stress by regulating reactive species production and enhancing the glutathione/oxidized glutathione ratio (Kim 2007; Sousa et al. 2018). Our observations indicate that crude extracts, with total phenol and flavonoid concentrations, exhibit increased antioxidant activity. Among the extracts from A. porphyrocarpa, the methanolic extract (MEAPL) is rich in phenolic compounds, while the chloroform extract (CEAPL) has a higher flavonoid content. DPPH and reducing power assays demonstrated that CEAPL possesses strong scavenging activities, with an IC50 value of 128.94 μg/mL, highlighting its superior antioxidant potential compared to other plant extracts. Overall, CEAPL emerges as a promising candidate for developing antioxidant compounds.

Thrombolysis is a technique aimed at enhancing blood flow, preventing organ and tissue damage, and dissolving blood clots (Rakib et al. 2020). Thrombolytic agents like streptokinase are utilized to break down clots. Typically, these agents activate plasminogen, converting it into plasmin, which acts to liquefy blood coagulates. Various plant sources, including seeds, leaves, herbs, and fruits, possess fibrinolytic properties and exhibit anti‐clotting and anti‐platelet effects (Fahad et al. 2021; Di Cera et al. 1997). Our screening revealed that all organic fractions of *A. porphyrocarpa* demonstrated significant clot lysis potential compared to saline water, suggesting its beneficial effects on coagulation disorders, with CFAPL exhibiting the strongest clot lysis properties among the extracts tested.

To predict the antioxidant, cytotoxic, thrombolytic, and neuropharmacological activities of MEAPL, a computer‐aided drug development tool has also been employed in addition to conventional laboratory techniques. The previously described creative strategies aim to simplify the intricate biological processes and connect pharmaceutical reactions with experimental data. Remarkably, computer‐aided methods have a significant role in driving advances in medicine and healthcare. They provide a way to obtain important insights with minimal resource expenditure, allowing thorough investigation of biological environments while maintaining the accuracy of observations (Pelkonen et al. 2011). Using this cutting‐edge computer‐aided approach, the existence of bioactive isolates was meticulously confirmed. It started with a complex combination of in‐depth literature reviews and GC–MS analysis. The ADME/T characteristics of the chosen bioactive isolates were the subject of the following study. To provide the greatest level of screening, the isolates were chosen for docking evaluation and carefully adhered to Lipinski's “Rules of Five”. The most fascinating compounds underwent extensive molecular docking to determine their binding affinity. The effectiveness of plant isolates selected according to a SAR technique and matching the SAR in the database was verified using the PASS software before the commencement of our investigation. Next, to anticipate ligand‐target interactions and reveal the binding patterns of active isolates against important enzymes, molecular docking—a potent method in computational biology and drug design—was utilized. This method supports the results of our experiments and clarifies possible biochemical routes and processes behind different pharmacological actions. To provide important insights into the mechanisms of action of bioactive isolates, our research adopted molecular docking analysis to identify the complex molecular bindings of these isolates to protein targets that are crucial in cytotoxic, antioxidant, thrombolytic, and neuropharmacological pathways. 2‐Pyridinecarboxylic acid exhibited significant docking value when interacting with 1DGH protein. These interactions involved specific amino acid sequences (TRP303, HIS194, HIS303, HIS135, ARG203, GIN442, VAL302, PRO151, PHE198, LYS237, ASN213), mediated through hydrogen bonds, van der Waals forces, and carbon‐hydrogen bonds. These interactions are attributed to the observed antioxidant activity (Xu et al. 2017). Cytotoxic behavior was exhibited by. propionic acid, 3‐(isobutylthio)‐ with the highest binding score among specified molecules by notable binding affinity toward ASP863, THR862, GLN799, LEU852, MET800, VAL734, ALA751, LYS753, LEY796, THR798, PHE864, ALA771, GLU770, and MET774 through conventional bonding and 2‐propanone, 1‐hydroxy‐ interacts with GLN64 for thrombolytic activity. 2‐Cyclobutene‐1‐carboxamide exhibits neuropharmacological activity by bonding with ASN208, and GLY207 for antidepressant effects and isobutyric acid, 2,2,2‐trichloroethyl ester binds with MET149, TRP81, ILF146, LEU92, PRO82, PHE83, ILE146, VAL87 for anxiolytic effects (Kumar et al. 2014).

The isolates under study not only passed Lipinski's “Rule of Five,” but they also showed encouraging drug‐like characteristics against thrombosis development, cytotoxicity, anxiety, and depression. The examined isolates satisfied all of Lipinski's requirements, which include molecular weight (< 500 g/mol), hydrogen bond acceptor (≤ 10), hydrogen bond donor (≤ 5), logP (< 5), and molar refractivity (40–130), Table S3. This rule of compliance implies good bioavailability. The study's results demonstrated that these isolates had a low number of rule violations (between 0 and 1), suggesting that they might be useful candidates for medication development to treat a variety of health conditions. Interestingly, in silico results confirmed the biological activities of the isolates by agreeing with experimental data. In addition, the study linked pharmacokinetic and pharmacodynamic characteristics to experimental results to provide pharmacological insights, opening the door for more research before clinical trials.

## 5 Conclusion

To summarize, the findings of this study demonstrate that the plant holds considerable promise as a source of therapeutic agents, with organic fractions showing strong activity against thrombosis and neuropsychiatric disorders. The anti‐oxidant efficacy of this plant further suggests a protective role against oxidative stress–related degenerative conditions. Moreover, molecular docking revealed strong binding affinities of selected compounds with relevant protein targets. Collectively, these results support the plant as a promising source for novel therapeutic agents with broad pharmacological applications. However, further studies are required to identify the specific secondary metabolites responsible for these effects and to elucidate their underlying mechanisms of action with more updated cytotoxicity tests.

## Author Contributions

J.H.A. and F.I.F., conducted the study and developed the design of this experiment. S.M.T., M.N.I. and S.A., set up the facilities for the experimental investigation and oversaw it. J.H.A., K.B., S.M.A.K.A., M.M.B, and R.K., prepared the extract preparation, data collection, investigational work and literature review. F.I.F., worked on statistical analysis with the study design and interpretation of the findings with S.M.T. and M.N.I. S.M.A.K.A., performs the computational analysis with the help of M.N.I. and S.M.T. J.H.A., F.I.F., M.M.B, F.T.R. and S.M.A.K.A., performed the initial drafting. S.M.T., M.N.I, S.R. and F.I.F, reviewed and updated the manuscript for any required modifications to the format. S.M.T. and M.N.I. supervised the entire research work. All authors went through and authorized the published format of the work.

## Ethics Statement

The authors have nothing to report.

## Conflicts of Interest

The authors declare no conflicts of interest.

## Supporting information


**Table S1:** GC–MS data and identified compounds from MEAPL (methanolic extract of *A. porphyrocarpa* leaves).
**Table S2:** Molecular docking scores of selected compounds from MEAPL with specific proteins.
**Table S3:** Lipinski's violations study for drug likeness of selected compounds from MEAPL.

## Data Availability

The data that support the findings of this study are available from the corresponding author upon reasonable request.
