# Supplementary material for: Multimodal Pharmacological Assessment of Arenga porphyrocarpa Palm Leaves and Stems Extracts: Insights From In Vivo, In Vitro, and In Silico Approaches
Source: Food Sci Nutr. 2025 Nov 19;13(11):e71178. doi: 10.1002/fsn3.71178 (PMC12628079; doi:10.1002/fsn3.71178)
Supplement: Supplementary file 1 — Table S1: GC–MS data and identified compounds from MEAPL (methanolic extract of A. porphyrocarpa leaves). Table S2: Molecular docking scores of selected compounds from MEAPL with specific proteins. Table S3: Lipinski's violations study for drug likeness of selected compounds from MEAPL. [file FSN3-13-e71178-s001.docx]

*Original research*

**Multimodal Pharmacological Assessment of *Arenga porphyrocarpa* Palm Leaves & Stems Extracts: Insights from *In-vivo*, *In-vitro*, and In-silico Approaches**

Jahid Hasan Azad^1, †^, Fowzul Islam Fahad^1, †^, Koushik Barua^1^, S. M. Asadul Karim Azad^1^, Safaet Alam^2,3^, Fahmida Tasnim Richi^2,4^, Rasel Khan^1^, Syed Mohammed Tareq^1^, Muhammad Mutasim Billah^1^, Md. Sohel Rana^5^, Mohammad Nazmul Islam^1, 5*^

^1^ Department of Pharmacy, International Islamic University Chittagong, Chittagong 4318, Bangladesh

^2^ Department of Pharmaceutical Chemistry, Faculty of Pharmacy, University of Dhaka, Dhaka-1000, Bangladesh

^3^ Chemical research division, BCSIR Dhaka Laboratories, Bangladesh Council of Scientific and Industrial Research, Dhaka-1205, Bangladesh

^4^ Department of Pharmacy, University of Asia Pacific, 74/A, Green Road, Farmgate, Dhaka-1215, Bangladesh

^5^ Department of Pharmacy, Jahangirnagar University, Savar, Dhaka-1342, Bangladesh

^†^ Authors contribution equally on this research.

**Corresponding authors:**

**Mohammad Nazmul Islam:**

Assistant Professor, Department of Pharmacy, International Islamic University Chittagong, Chittagong 4318, Bangladesh; E-mail: nazmul@iiuc.ac.bd

**Table S1:** GC-MS data and identified compounds from MEAPL (methanolic extract of *A. porphyrocarpa* leaves).

| **Sl. No** | **Compound Name** | **Chemical Formula** | **MW**  **(g/mol)** | **RT** | **Area** | **Nature** |
| --- | --- | --- | --- | --- | --- | --- |
| 01 | Methyl Alcohol | CH_4_O | 32.04 | 3.56 | 13.92 | Alcohol |
| 02 | Propanoic acid, 2-hydroxy-, methyl ester, (.+/-.)- | C_4_H_8_O_3_ | 104.11 | 4.203 | 12.47 | Fatty Acid |
| 03 | 3-Hexanone | C_6_H_12_O | 100.16 | 4.282 | 5.49 | ketone |
| 04 | Propionic acid, 3-(isobutylthio)- | C_7_H_14_O_2_S | 162.25 | 4.34 | 7.94 | Fatty Acid |
| 05 | 2-Pyridinecarboxylic acid | C_6_H_5_NO_2_ | 123.11 | 4.395 | 2.54 | Carboxylic acid. |
| 06 | 4-Hydroxybutyric acid hydrazide | C_4_H_10_N_2_O_2_ | 118.13 | 4.525 | 1.18 | Carbohydrazide |
| 07 | 2-Propanone, 1-hydroxy- | C_3_H_6_O_2_ | 74.08 | 4.689 | 0.82 | Alpha-hydroxy ketone |
| 08 | 3-Ethoxy-1,2-propanediol | C_5_H_12_O_3_ | 120.15 | 5.134 | 0.54 | Secondary alcohol |
| 09 | Isobutyric acid, 2,2,2-trichloroethyl ester | C_6_H_9_Cl_3_O_2_ | 219.49 | 5.18 | 0.50 | Ester |
| 10 | 3,3-Dimethoxy-2-butanone | C_6_H_12_O_3_ | 132.16 | 5.262 | 2.90 | Ketone |
| 11 | Butyl 2,5,8,11-tetraoxatridecan-13-oate | C_13_H_26_O_6_ | 278.34 | 5.348 | 1.32 | Butyl ester |
| 12 | Dimethyl Sulfoxide | (CH_3_)_2_SO | 78.14 | 5.679 | 0.59 | Polar aprotic solvent |
| 13 | Sulfuric acid, dimethyl ester | C_2_H_6_O_4_S | 126.13 | 5.745 | 0.50 | Ester |
| 14 | 4-Oxo-5-methoxycarbonylthio-3-thiopentanoic acid, methyl ester | C₆H₈O₃S₂ | 192.26 | 5.796 | 0.50 | Ester |
| 15 | 2-Cyclobutene-1-carboxamide | C_5_H_7_NO | 97.117 | 6.75 | 0.36 | Cyclic carboxamide |
| 16 | 1-(2-Methylbutoxy)-7-pentyl-2,2,4,4,6,6-hexamethyl-1,3,5,7-tetraoxa-2,4,6-trisilaheptane | C_16_H_40_O_4_Si_3_ | 380.743 | 7.43 | 0.45 | Siloxane compound |
| 17 | 3,3-Diisopropoxy-1,1,1,5,5,5-hexamethyltrisiloxane | C_12_H_32_O_4_Si_3_ | 324.63 | 7.678 | 0.86 | Siloxane compound |
| 18 | Oxirane, 2-[(nonyloxy)methyl]- | C_12_H_24_O_2_ | 200.32 | 9.646 | 0.45 | Alkane |
| 19 | 1-Decanol | CH_3_(CH_2_)_9_OH | 158.28 | 12.1 | 0.41 | Primary alcohol |
| 20 | Bis(heptamethylcyclotetrasiloxy)siloxane | C_14_H_42_O_9_Si_8_ | 579.2 | 12.48 | 1.04 | Siloxane compound |
| 21 | Decane, 1-iodo- | C_10_H_21_I | 268.18 | 12.525 | 0.32 | haloalkane |
| 22 | Benzeneacetic acid, 3-tetradecyl ester | C_22_H_36_O_2_ | 332.5 | 13.295 | 0.32 | Fatty Acid |
| 23 | Nonane, 3-methyl-5-propyl- | C_13_H_28_ | 184.36 | 13.588 | 0.59 | Alkane |
| 24 | Benzene, (1-butylheptyl)- | C_17_H_28_ | 232.40 | 14.004 | 0.63 | Aromatic compound |
| 25 | Hexadecane | C_16_H_34_ | 226.44 | 14.07 | 0.36 | Alkane |
| 26 | Benzene, (1-propyloctyl)- | C_17_H_28_ | 232.40 | 14.123 | 0.32 | Aromatic compound |
| 27 | Octadecane-1,2-diol, 2TMS derivative | C_24_H_54_O_2_Si_2_ | 430.9 | 14.351 | 0.54 | Glycol |
| 28 | Eicosane | C_20_H_42_ | 282.55 | 14.703 | 1.09 | Alkane |
| 29 | Hydratropic acid, undec-2-en-1-yl ester | C_20_H_30_O_2_ | 302.451 | 14.864 | 0.45 | Ester |
| 30 | Benzene, (1-pentylheptyl)- | C_18_H_30_ | 246.43 | 15.113 | 0.68 | Alkene |
| 31 | Benzene, (1-butyloctyl)- | C_18_H_30_ | 246.43 | 15.175 | 0.45 | Alkene |
| 32 | Neophytadiene | C_20_H_38_ | 278.51 | 16.524 | 1.45 | Sesquiterpenoid |
| 33 | Hexadecanoic acid, methyl ester | C_17_H_34_O_2_ | 270.45 | 17.843 | 5.58 | Ester |
| 34 | Dibutyl phthalate | C_16_H_22_O_4_ | 278.34 | 18.438 | 0.54 | Anhydride |
| 35 | Diethylene glycol monododecyl ether | C_16_H_34_O_3_ | 274.44 | 18.966 | 0.36 | Ether |
| 36 | 9,12-Octadecadienoic acid (Z,Z)-, methyl ester | C_19_H_34_O_2_ | 294.47 | 20.575 | 3.22 | Ester |
| 37 | 6-Octadecenoic acid, methyl ester, (Z)- | C_19_H_36_O_2_ | 296.49 | 20.676 | 6.39 | Ester |
| 38 | (Z)-15-Octadecenoic acid methyl ester | C_19_H_36_O_2_ | 296.5 | 20.765 | 0.41 | Ester |
| 39 | Phytol | C_20_H_40_O | 296.53 | 20.828 | 1.50 | Diterpene |
| 40 | Methyl stearate | C_19_H_38_O_2_ | 298.50 | 21.085 | 1.68 | Ester |
| 41 | Tetradecanamide | C_14_H_29_NO | 227.37 | 22.03 | 0.45 | Amide |
| 42 | 9-Octadecenamide, (Z)- | C_18_H_35_NO | 281.48 | 25.072 | 11.79 | Amide |
| 43 | Ethyl 2-formyl-1-cyclopropanecarboxylate, trans | C_7_H_10_O_3_ | 142.15 | 25.265 | 0.86 | Ester |
| 44 | Oxiraneundecanoic acid, 3-pentyl-, methyl ester, trans- | C_19_H_36_O_3_ | 312.49 | 30.773 | 0.32 | Ester |
| 45 | 3-(4-Bromophenyl)-7-hydroxy-4H-chromen-4-one, TMS derivative | C_15_H_9_BrO_3_ | 316.90 | 31.005 | 0.36 | Enone |
| 46 | Squalene | C_30_H_50_ | 410.7 | 31.999 | 2.27 | Triterpene |
| 47 | 1,3,5,7-Tetramethyl-3,5,7-triphenyl-1-[2-(1,3,3,5,5-pentamethyl-2,4,6-trioxa-trisilacyclohexyl)ethyl]-2,4,6,8-tetraoxa-1,3,5,7- | C_29_H_46_O_7_Si_7_ | 703.3 | 32.183 | 0.50 | Poly-siloxane |
| 48 | Bis[di(trimethylsiloxy)phenylsiloxy]trimethylsiloxyphenylsiloxane | C_33_H_60_O_7_Si_8_ | 793.5 | 33.646 | 0.41 | Siloxane compound |
| 49 | 2-Methyl-6-(5-methyl-2-thiazolin-2-ylamino)pyridine | C_10_H_13_N_3_S | 207.3 | 35.194 | 0.41 | Aromatic heterocyclic compound |
| 50 | Cyclohexene, 1-nonyl- | C_15_H_28_ | 208.38 | 35.648 | 0.50 | Cyclic alkene |
| 51 | Vitamin E | C_29_H_50_O_2_ | 430.706 | 36.564 | 1.36 | Alpha-tocopherol |

**Table S2:** Molecular docking scores of selected compounds from MEAPL with specific proteins.

| **Sl. No** | **Compound Name** | **Antidepressant** | **Anti-oxidant** | **Anxiolytic** | **Thrombolytic** | **Cytotoxic** |
| --- | --- | --- | --- | --- | --- | --- |
|  |  | **6VRH** | **1DGH** | **3U5K** | **1A5H** | **3PP0** |
| 01 | Methyl Alcohol | -2.051 | -2.153 | -2.474 | -2.858 | -1.213 |
| 02 | Propanoic acid, 2-hydroxy-, methyl ester, (.+/-.)- | -2.836 | -3.56 | -3.563 | -3.458 | -2.635 |
| 03 | 3-Hexanone | -3.071 | -3.879 | -3.842 | -4.499 | -3.098 |
| 04 | Propionic acid, 3-(isobutylthio)- | -2.141 | -7.271 | -4.181 | -3.499 | -8.825 |
| 05 | 2-Pyridinecarboxylic acid | -3.022 | -7.546 | -4.787 | -4.206 | -8.436 |
| 06 | 4-Hydroxybutyric acid hydrazide | -2.786 | -4.72 | -3.301 | -3.317 | -3.205 |
| 07 | 2-Propanone, 1-hydroxy- | -2.92 | -3.227 | -4.551 | -4.903 | -2.528 |
| 08 | 3-Ethoxy-1,2-propanediol | -3.081 | -4.489 | -5.091 | -3.909 | -3.671 |
| 09 | Isobutyric acid, 2,2,2-trichloroethyl ester | -3.553 | -4.311 | -5.333 | -4.138 | -4.407 |
| 10 | 3,3-Dimethoxy-2-butanone | -3.098 | -3.073 | -4.275 | -3.736 | -2.497 |
| 11 | Butyl 2,5,8,11-tetraoxatridecan-13-oate | -2.987 | -6.117 | -2.969 | -4.121 | -5.554 |
| 12 | Dimethyl Sulfoxide | -2.594 | -2.443 | -3.001 | -3.942 | -1.603 |
| 13 | Sulfuric acid, dimethyl ester | -2.343 | -2.176 | -3.072 | -2.723 | -1.746 |
| 14 | 2-Cyclobutene-1-carboxamide | -3.781 | -4.152 | -5.041 | -4.234 | -3.74 |
| 15 | 1-(2-Methylbutoxy)-7-pentyl-2,2,4,4,6,6-hexamethyl-1,3,5,7-tetraoxa-2,4,6-trisilaheptane | -3.204 | 0.299 | -4.452 | -2.833 | -3.485 |
| 16 | Diazepam | -6.5 | -4.123 | -6.851 | -6.966 | -2.268 |
| 17 | Ascorbic acid | -4.74 | -6.928 | -5.794 | -5.651 | -8.25 |
| 18 | Fluoxetine HCl | -9.863 | -6.185 | -9.435 | -8.027 | -4.384 |
| 19 | Streptokinase | -5.191 | -7.15 | -4.484 | -5.535 | -9.585 |
| 20 | Vincristine Sulphate | 0 | 0 | -2.187 | -1.354 | -1.094 |

MEAPL: Methanolic extract of *A. porphyrocarpa* leaves

**Table S3:** Lipinski’s violations study for drug likeness of of selected compounds from MEAPL.

| **Sl. No** | **Compound Name** | **MW** | **HBA** | **HBD** | **AMR** | **TPSA** | **LogP** | **Lipinski’s Violations** |
| --- | --- | --- | --- | --- | --- | --- | --- | --- |
| 01 | Methyl Alcohol | 32.042g/mol | 1 | 1 | 8.08 | 20.23 Å² | -0.28 | 0 |
| 02 | Propanoic acid, 2-hydroxy-, methyl ester, (.+/-.)- | 104.1g/mol | 3 | 1 | 23.79 | 46.53 Å² | 0.01 | 0 |
| 03 | 3-Hexanone | 100.16g/mol | 1 | 0 | 31.16 | 17.07 Å² | 1.54 | 0 |
| 04 | Propionic acid, 3-(isobutylthio)- | 162.25g/mol | 2 | 1 | 45.13 | 62.60 Å² | 1.65 | 0 |
| 05 | 2-Pyridinecarboxylic acid | 123.11g/mol | 3 | 1 | 31.2 | 50.19 Å² | 0.37 | 0 |
| 06 | 4-Hydroxybutyric acid hydrazide | 118.13g/mol | 3 | 3 | 28.21 | 75.35 Å² | -1.04 | 0 |
| 07 | 2-Propanone, 1-hydroxy- | 74.08g/mol | 2 | 1 | 17.9 | 37.30 Å² | -0.25 | 0 |
| 08 | 3-Ethoxy-1,2-propanediol | 120.15g/mol | 3 | 2 | 29.56 | 49.69 Å² | -0.12 | 0 |
| 09 | Isobutyric acid, 2,2,2-trichloroethyl ester | 219.5g/mol | 2 | 0 | 46.67 | 26.30 Å² | 2.54 | 0 |
| 10 | 3,3-Dimethoxy-2-butanone | 132.16g/mol | 3 | 0 | 33.36 | 35.53 Å² | 0.51 | 0 |
| 11 | Butyl 2,5,8,11-tetraoxatridecan-13-oate | 278.34g/mol | 6 | 0 | 70.23 | 63.22 Å² | 1.61 | 0 |
| 12 | Dimethyl Sulfoxide | 78.14g/mol | 1 | 0 | 20.01 | 36.28 Å² | 0.05 | 0 |
| 13 | Sulfuric acid, dimethyl ester | 126.13g/mol | 4 | 0 | 22.86 | 60.98 Å² | 0.01 | 0 |
| 14 | 2-Cyclobutene-1-carboxamide | 97.12g/mol | 1 | 1 | 26.47 | 43.09 Å² | 0.21 | 0 |
| 15 | 1-(2-Methylbutoxy)-7-pentyl-2,2,4,4,6,6-hexamethyl-1,3,5,7-tetraoxa-2,4,6-trisilaheptane | 380.74g/mol | 4 | 0 | 106.97 | 36.92 Å² | 3.85 | 0 |

MEAPL: Methanolic extract of *A. porphyrocarpa* leaves
